# Long-term mortality and time to minimally invasive puncture and drainage in spontaneous intracerebral hemorrhage

**DOI:** 10.3389/fneur.2026.1829539

**Published:** 2026-04-29

**Authors:** Nan Gan, Qiyu Li, Jinrong Hu, Jian Liu, Xinyue Zheng, Xupeng Li, Jian Miao, Tao Ke

**Affiliations:** 1 Department of Microbiology and Immunology, Basic Medicine College, Jinan University, Guangzhou, China; 2 Department of Neurology, Xianyang Hospital of Yan’an University, Xianyang, China; 3 Department of Systems Biomedical Science, School of Medicine, Jinan University, Guangzhou, China; 4 Department of Neurology, The First Affiliated Hospital of Jinan University, Guangzhou, China; 5 Department of Neurosurgery, Yan’an University Second Affiliated Hospital, Yulin, China; 6 Department of Neurology, Shaanxi University of Chinese Medicine Second Affiliated Hospital, Xianyang, China; 7 Department of Basic Medicine, School of Capital Medical University, Beijing, China; 8 Department of Neurology, School of Biological Science and Medical Engineering, Beihang University, Beijing, China

**Keywords:** hematoma volume, minimally invasive puncture and drainage, mortality, spontaneous intracerebral hemorrhage, time to evacuation

## Abstract

**Objectives:**

Minimally invasive puncture and drainage (MIPD) is a safe and effective treatment for spontaneous intracerebral hemorrhage (sICH). However, the impact of time to evacuation on clinical outcomes remains unclear. This study aims to assess the association between the time from symptom onset to MIPD and long-term mortality.

**Methods:**

The study retrospectively included consecutive patients with a hematoma ≥ 20 mL who underwent MIPD within 24 h of symptom onset. Patients were stratified by different time windows (0–6 h, 6–12 h, and 12–24 h) from symptom onset to MIPD. One-year (long-term) mortality was defined as the primary outcome. Secondary outcomes included the incidence of rebleeding, the Glasgow Coma Scale (GCS) score at discharge, and modified Rankin Scale (mRS) scores at 3 and 6 months. The association between time to evacuation and clinical outcomes was assessed using multivariate logistic regression and inverse probability of treatment weighting (IPTW) analysis.

**Results:**

A total of 214 eligible patients were included in our study. Patients who underwent MIPD within 6 h or 6 to 12 h had a higher long-term mortality rate compared to those treated within 12 to 24 h (48.48, 50.56, and 30.34%, respectively; *p* = 0.02). In IPTW analysis, undergoing MIPD within 12–24 h of symptom onset was associated with reduced short-term mortality [odds ratio (OR), 0.519; 95%CI (0.290–0.929), *p* = 0.03] and long-term mortality [OR, 0.530; 95% CI (0.300–0.937), *p* = 0.03].

**Conclusion:**

In patients with sICH, the time to MIPD within 12 to 24 h was associated with a decreased risk of long-term mortality.

## Introduction

Spontaneous intracerebral hemorrhage (sICH) accounts for 10–15% of all strokes worldwide each year (1). Despite receiving systematic care, patients with sICH experience high morbidity and a mortality rate of approximately 30–40% (2). Efficient treatment focuses on early hematoma evacuation and edema reduction, including craniotomy, conservative treatment, and minimally invasive puncture and drainage (MIPD) in many countries (3–5). Minimally invasive surgical evacuation procedures has been investigated as alternatives to conventional craniotomy (6). Numerous clinical trials have assessed the impact of minimally invasive evacuation on sICH outcomes. The MISTIE III trial, which investigated minimally invasive catheter evacuation followed by thrombolysis, found lower one-year mortality in the minimally invasive group compared to the standard treatment group (7). A prospective non-randomized comparative study also supported the result that patients undergoing minimally invasive evacuation had higher rates of neurological independence and lower one-year mortality (8). Additionally, the ENRICH trial demonstrated that minimally invasive hematoma evacuation resulted in better functional outcomes at 6 months when combined with guideline-based medical management (9). Overall, minimally invasive evacuation seems to be a safe and effective treatment for sICH.

Previous clinical trials have formulated different inclusion criteria regarding time windows, with most participants randomized within 24 h of symptom onset (7–9). However, the STICH II trial, which compared early surgery versus initial conservative treatment in patients with spontaneous supratentorial lobar intracerebral hematomas, found that early surgery (within 12 h) did not increase mortality or disability at 6 months (10). One retrospective study found that patients who underwent MIPD within 6 h had a higher mortality rate at 3 months compared to those treated later (11). However, the impact of different time windows for MIPD remains uncertain in patients with sICH. To better improve clinical prognosis in MIPD for sICH, further evidence on the relationship between time to evacuation and prognosis is needed.

In this study, we retrospectively collected data on patients who underwent MIPD from two centers, aiming to investigate the association between time to evacuation and clinical outcomes. Additionally, we compared one-year mortality rates across different time intervals.

## Method

### Study population

We retrospectively reviewed electronic medical records of patients with sICH from the First Hospital of Yulin and Xianyang Hospital of Yan’an University, covering the period from August 1, 2019, to September 18, 2023. The study adhered to the principles outlined in the Declaration of Helsinki and was approved by the Ethics Committee of the First Hospital of Yulin. Patients were included based on the following criteria: (1) age > 18 years; (2) time from symptom onset to MIPD within 24 h; (3) Glasgow Coma Scale (GCS) score ≥ 5 and modified Rankin Scale (mRS) score ≤ 1 prior to sICH; (4) hematoma volume ≥ 20 mL as assessed by CT scan. Patients were excluded if they had hemorrhage resulting from trauma, ruptured aneurysm, cerebral arteriovenous malformation, or vascular anomalies. Additional exclusion criteria included coagulation disorders, cerebral tumors, intracranial infections, brainstem failure at admission, severe complications, or pregnancy.

We recorded the population characteristics and clinical data of patients, including age, sex, medical history (hypertension, diabetes, coronary heart disease), current smoking status, GCS score at admission, hemorrhage volume (calculated using the Coniglobus formula: A × B × C × 1/2) (12), systolic and diastolic blood pressure at admission, time from symptom onset to admission, and the surgical procedure performed.

### Procedures

The surgery was performed by a well-trained surgical team. In both participating centers, MIPD was performed according to similar institutional principles regarding patient selection, preoperative assessment, hematoma drainage, and postoperative management. Puncture site was selected based on the maximum cross-sectional area of the hematoma on preoperative CT scans, while avoiding critical functional areas and major vessels. Under local anesthesia, a suitable YL-I puncture needle was used to access the hematoma center, where a drainage catheter was placed. A 10-ml syringe was connected to the catheter and used to aspirate uncoagulated blood until no further fluid was obtained. Urokinase (10,000 U) mixed with 5 mL of saline was infused into the hematoma cavity and allowed to react for 1 h before being drained into a closed extracranial drainage system. CT scans were obtained on the 1st, 3rd, 5th, and 7th days post-operation. Urokinase/saline infusion was repeated until the drainage catheter was removed, which was determined based on the remaining hematoma volume of less than 10 mL on follow-up CT scans and the patient’s stable condition.

### Clinical outcomes

Patients’ clinical prognosis was assessed through telephone interviews. The functional recovery of patients who underwent MIPD was evaluated using the mRS score. The primary outcome was mortality within 1 year, defined as a mRS score of 6. Secondary outcomes included the rate of rebleeding, GCS score at discharge, as well as favorable and excellent outcomes assessed at 1, 3, 6, and 12 months. In this study, a favorable outcome was defined as an mRS score of 0 to 2, and an excellent outcome as a score of 0 to 1.

### Statistical analysis

Baseline population characteristics and clinical outcomes were described across three groups (0–6 h, 6–12 h, and 12–24 h time to evacuation) using medians and the 25th-75th percentiles for continuous variables, and frequencies and percentages for categorical variables. The differences between categorical and continuous variables were analyzed using the Chi-square test and the Kruskal–Wallis H test, respectively.

As a secondary analysis, the time to evacuation was further divided into 0–12 h and 12–24 h groups. The association between clinical prognosis and time to evacuation was assessed using a logistic regression model. In the multivariate logistic regression, we adjusted for variables that could plausibly confound the relationship between time to evacuation and clinical outcomes, including age, sex, hypertension, GCS at admission, hematoma volume, coronary heart disease, surgery procedure time, and site of hematoma. Additionally, an inverse probability treatment weighting (IPTW) analysis was conducted, incorporating age, sex, hypertension, GCS at admission, hematoma volume, coronary heart disease, surgery procedure time, and site of hematoma into the model. Restricted cubic splines were used to display the odds ratios (ORs) and 95% confidence intervals (CIs) for the association between time to evacuation and mortality within 1 year. Kaplan–Meier Survival curve was utilized to explore the differences in mortality risk among different time to evacuation intervals. Finally, we assessed the effects of time from onset to operation on one-year mortality across different cohorts, including GCS score at admission (0–9 versus 9–15), hematoma volume (20–40 mL versus ≥ 40 mL), and site of hematoma, based on IPTW model.

The significance threshold for statistical testing was set at *p* < 0.05 (two-tailed). All statistical analyses were performed using IBM SPSS Statistics (Version 26.0 program. Armonk, NY: IBM Corp) and R software (version 1.4.1106).

## Result

### Baseline characteristics of patients stratified by time to evacuation

A total of 214 patients who received MIPD within 24 h were enrolled in our study. The median age was 64 years, and 136 (63.55%) were men. Regarding the time from symptom onset to MIPD, 35 patients (16.35%) underwent surgery within 6 h, 90 patients (42.06%) between 6 and 12 h, and 89 patients (41.59%) underwent MIPD between 12 and 24 h after symptom onset (Table 1). The median hematoma volume was significantly larger in patients undergoing MIPD between 12 and 24 h after sICH than in the 0–6 h and 6–12 h groups (49 mL vs. 38 mL and 35 mL, respectively; *p* = 0.002). Additionally, hematoma volumes exceeding 40 mL were more frequent in the 12–24 h group. The median surgery procedure times for the 0–6 h, 6–12 h, and 12–24 h groups were 60, 38, and 30 min, respectively.

**Table 1:** Baseline characteristics.

Demographics	6 h (*n* = 35)	6–12 h (*n* = 90)	12–24 h (*n* = 89)	*p* value
Age, y, median (IQR)	65 (50–73)	63 (56–69)	64 (57–71)	0.68
Male, *n* (%)	18 (51.43)	58 (64.44)	60 (67.42)	0.24
Disease history, *n* (%)				
Hypertension	28 (80.00)	68 (75.56)	63 (70.79)	0.54
Diabetes	5 (14.29)	17 (18.89)	9 (10.11)	0.25
Coronary heart disease	6 (17.14)	8 (8.89)	2 (2.25)	0.01*
Current smoking	2 (5.71)	12 (13.33)	13 (14.61)	0.44*
Clinical features				
GCS score at admission, median (IQR)	6 (3–10)	8 (5–11)	8 (7–11)	0.01
GCS score at admission degree, *n* (%)				0.51
3–8	24 (68.57)	49 (54.44)	46 (51.69)	
9–12	8 (22.86)	27 (30.00)	27 (30.34)	
13–15	3 (8.57)	14 (15.56)	16 (17.98)	
Hematoma volume, median (IQR)	38 (25–52)	35 (27–45)	49 (30–60)	0.002
Hematoma volume ≥40 (ml)	16 (45.71)	35 (38.89)	55 (61.80)	0.01
Blood pressure at admission, median (IQR)				
Systolic BP (mm Hg)	170 (160–200)	175 (155–190)	168 (154–186)	0.27
Diastolic BP (mm Hg)	94 (90–102)	97 (88–108)	94 (85–104)	0.47
Site of hematoma				<0.001*
Basal ganglia	21 (60.00)	39 (43.33)	51 (57.30)	
Lobar	0	4 (4.44)	12 (13.48)	
Thalamus	10 (28.57)	43 (47.78)	23 (25.84)	
Brainstem	4 (11.43)	1 (1.11)	0	
Other	0	3 (3.33)	3 (3.37)	
Time variables, median (IQR)				
Time from onset to admission(h)	2 (2–2)	3 (3–4)	5 (3–6)	<0.001
Time from admission to treatment(h)	3 (2–4)	6 (5–7)	12 (9–14)	<0.001
Surgery procedure during time(m)	60 (30–120)	38 (30–55)	30 (25–40)	<0.001

GCS, Glasgow Coma Scale; BP, blood pressure; and IQR, interquartile range. *Indicated the comparison among three groups using Fisher’s exact test.

### Association of time to evacuation with clinical outcomes

**Figure 1:**
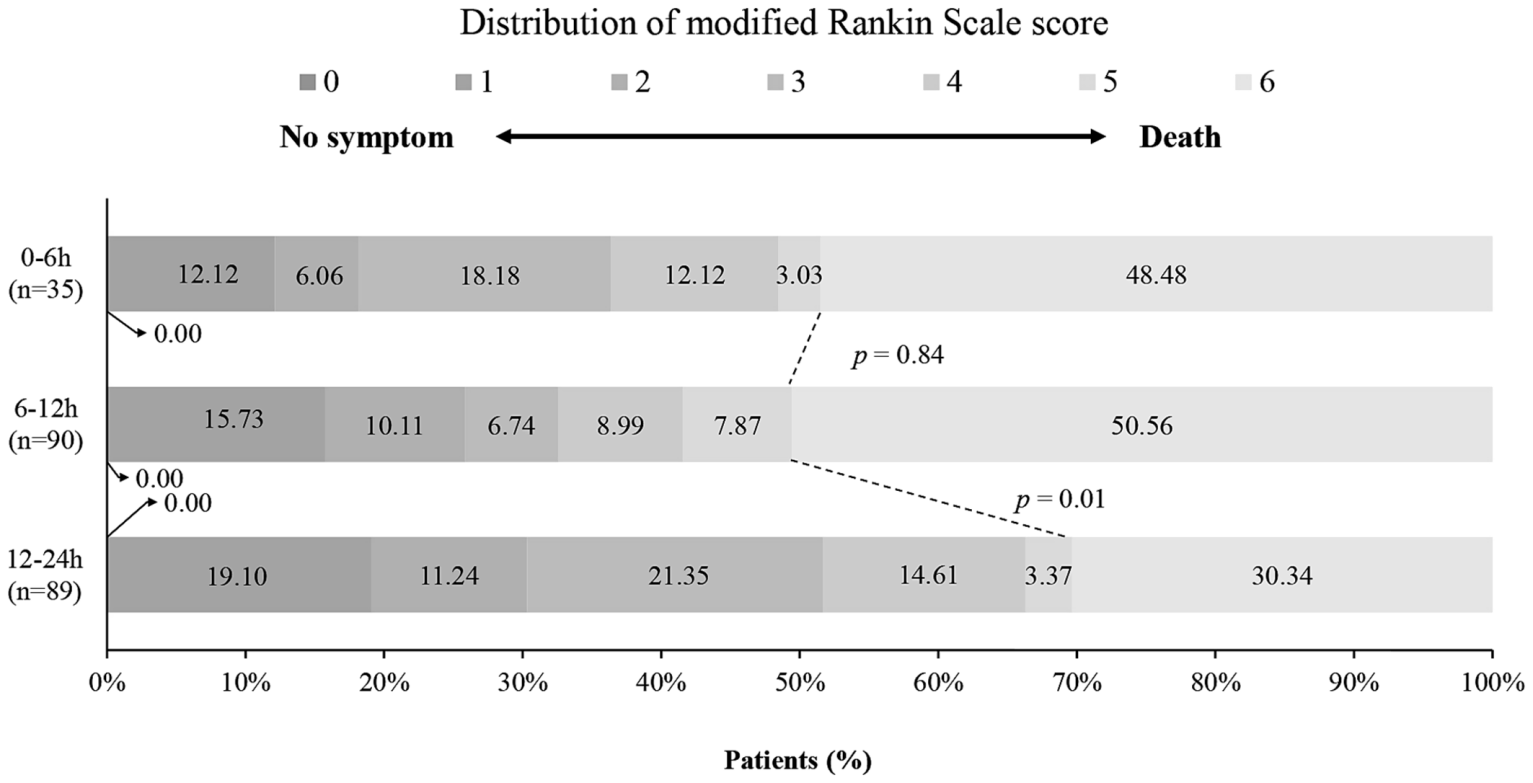
Distribution of the time to MIPD after symptom onset, as assessed by the mRS score at 1-year follow-up. The distribution of disability scores on the mRS score at 1 year is shown for three different time windows. MIPD refers to minimal invasive puncture and drainage; and mRS stands for Modified Rankin Scale.

**Figure 2:**
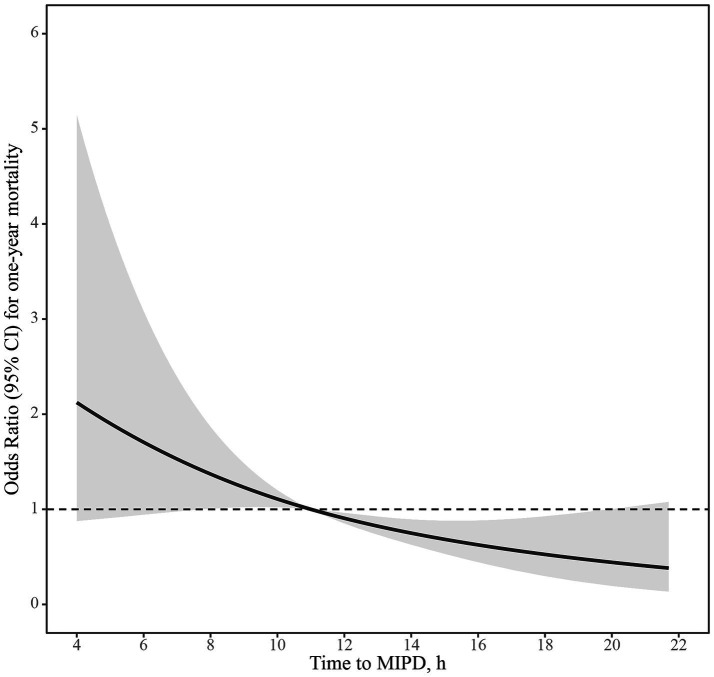
Odds ratio for one-year mortality based on the time to evacuation in patients who underwent MIPD. A restricted cubic spline curve illustrates the OR for mortality relative to the time from symptom onset to MIPD. MIPD refers to minimally invasive puncture and drainage.

The distribution of mRS scores at 1 year, stratified by the three groups, is shown in Figure 1. The frequencies of each outcome in the 0–6 h, 6–12 h, and 12–24 h groups are presented in Table 2. Patients who received MIPD within 6 h or between 6 to 12 h had a higher proportion of mortality compared to the 12–24 h group at 3, 6, and 12 months (3 months: 45.45% vs. 46.07% vs. 26.97%, *p* = 0.02; 6 months: 45.45% vs. 49.44% vs. 29.21%, *p* = 0.02; 1 year: 48.48% vs. 50.56% vs. 30.34%, *p* = 0.02). Figure 2 displays the time-dependent odds ratios (ORs) for mortality based on the time from onset to MIPD. A trend was observed where mortality decreased with prolonged time, with a sharp decrease occurring around 12 h. For secondary outcomes, there were no significant differences in rebleeding rates among the three cohorts (5.71% for 0–6 h, 5.56% for 6–12 h, and 2.25% for 12–24 h). For other secondary outcomes, no significant differences were identified among the groups in terms of GCS score at discharge, short- and long-term favorable outcomes, or short- and long-term excellent outcomes.

**Table 2:** Short- and long-term clinical outcomes stratified by three different time windows.

Clinical outcome	6 h (*n* = 35)	6–12 h (*n* = 90)	12–24 h (*n* = 89)	*p* value
Rebleeding, *n* (%)	2 (5.71)	5 (5.56)	2 (2.25)	0.49*
GCS score at discharge, median (IQR)	9 (10–14)	9 (10–14)	10 (12–15)	0.07
GCS score at discharge degree, *n* (%)				0.20
3–8	15 (42.86)	40 (44.44)	25 (28.09)	
9–12	7 (20.00)	19 (21.11)	21 (23.60)	
13–15	13 (37.14)	31 (34.44)	43 (48.31)	
mRS score at discharge, median (IQR)	4 (4–5)	4 (3–5)	4 (3–5)	0.35
mRS 0–1, *n* (%)	0	2 (2.22)	2 (2.25)	>0.99
mRS 0–2, *n* (%)	0	7 (7.78)	6 (6.74)	0.26
Mortality in hospital, *n* (%)	5 (14.29)	7 (7.78)	4 (4.49)	0.20*
mRS score at 3 months, median (IQR)	5 (4–6)	5 (3–6)	4 (3–6)	0.04
mRS 0–1 at 3 months, *n* (%)	0	5 (5.56)	4 (4.49)	0.59*
mRS 0–2 at 3 months, *n* (%)	4 (11.43)	17 (18.89)	16 (17.98)	0.60
Mortality within 3 months, *n* (%)	15 (45.45)	41 (46.07)	24 (26.97)	0.02
mRS score at 6 months, median (IQR)	5 (3–6)	5 (3–6)	4 (2–6)	0.04
mRS 0–1 at 6 months, *n* (%)	4 (11.43)	13 (14.44)	15 (16.85)	0.74
mRS 0–2 at 6 months, *n* (%)	6 (17.14)	21 (23.33)	24 (26.97)	0.51
Mortality within 6 months, *n* (%)	15 (45.45)	44 (49.44)	26 (29.21)	0.02
mRS score at 1 year, median (IQR)	5 (3–6)	6 (2–6)	3 (2–6)	0.03
mRS 0–1 at 1 year, *n* (%)	4 (11.43)	14 (15.56)	17 (19.10)	0.56
mRS 0–2 at 1 year, *n* (%)	6 (17.14)	23 (25.56)	27 (30.34)	0.32
Mortality within 1 year, *n* (%)	16 (48.48)	45 (50.56)	27 (30.34)	0.02

GCS, Glasgow Coma Scale; mRS, Modified Rankin Scale; and IQR, interquartile range. *Indicated the comparison among three groups using Fisher’s exact test.

**Figure 3:**
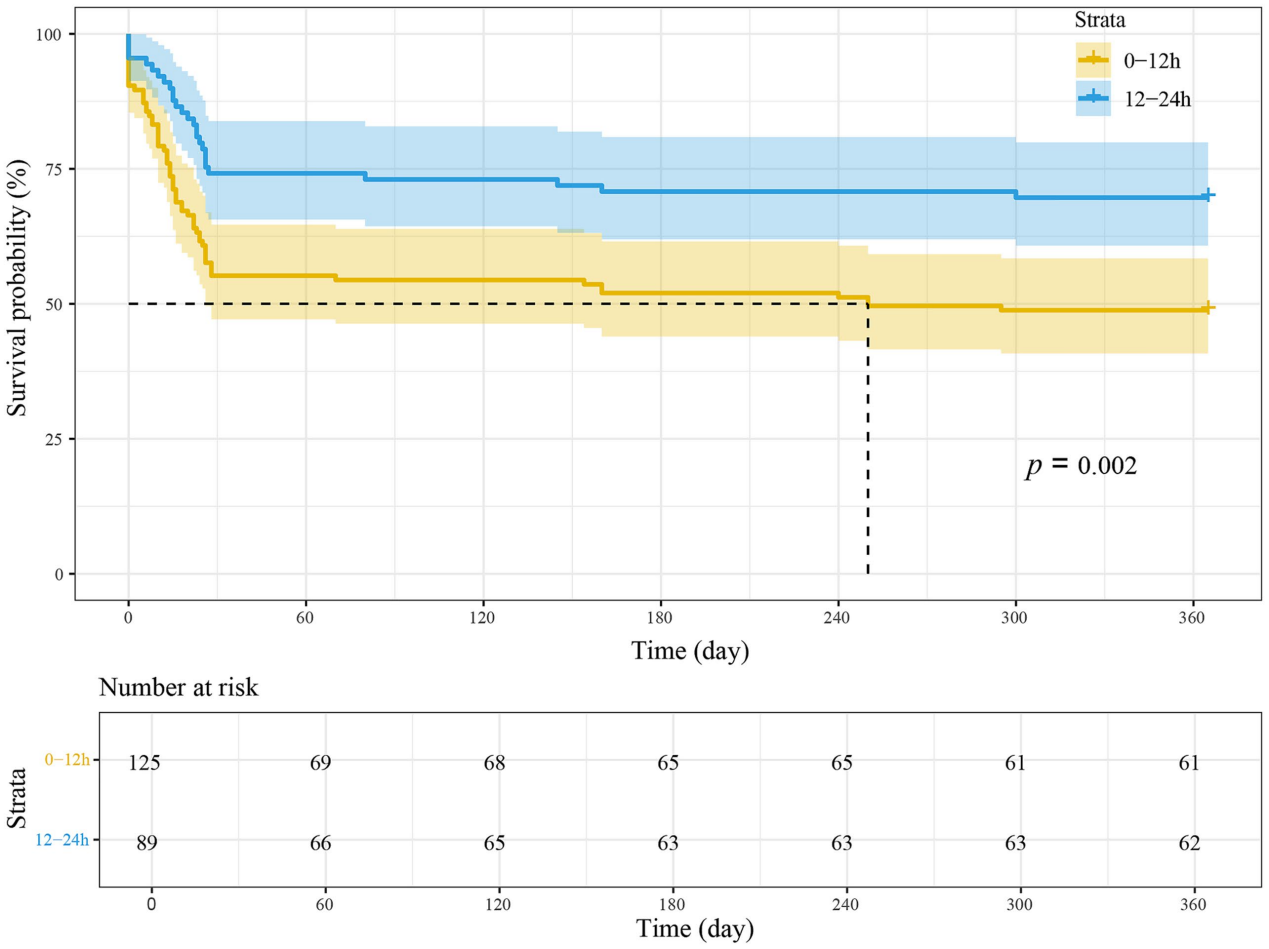
Kaplan–Meier survival analysis curves for 1-year mortality in different time to MIPD groups. Survival outcomes following MIPD were evaluated using the Kaplan–Meier method, with patients stratified according to the time to evacuation. MIPD refers to minimally invasive puncture and drainage.

Furthermore, patients were reanalyzed in two groups based on time to evacuation, with 12 h set as the threshold from symptom onset to MIPD. Receiving MIPD within 12 h after symptom onset did not increase the rebleeding rate compared to the group treated after 12 h (5.60% vs. 2.25%, *p* = 0.20). Additionally, there were no significant differences between the 0–12 h and 12–24 h groups in terms of favorable and excellent outcomes at 3 months, 6 months, and 1 year (Table 3). Kaplan–Meier analysis showed that patients in the 0–12 h group had a significantly lower cumulative survival probability than those in the 12–24 h group during follow-up, suggesting a higher mortality risk in the early-treatment group (log-rank *p* = 0.002, in Figure 3). Compared with the 12–24 h group, a higher mortality rate at 1 year was observed in the 0–12 h group (50.00% vs. 30.34%, *p* = 0.004). This difference was statistically significant in multivariable regression analyses [adjusted odds ratios (aOR), 0.451; 95% CI (0.226–0.901), *p* = 0.02] and IPTW analyses [odds ratio (OR), 0.530; 95% CI (0.300–0.937), *p* = 0.03].

**Table 3:** Association of time to evacuation with clinical outcomes.

Clinical outcome	<12 h (*n* = 125)	12–24 h (*n* = 89)	*p* value	Adjusted model		IPTW model	
				OR (95% CI)	*p* value	OR (95% CI)	*p* value
Rebleeding, *n* (%)	7 (5.60)	2 (2.25)	0.20	0.743 (0.134–4.130)^c^	0.73	0.694 (0.166–2.900)^c^	0.62
GCS score at discharge, median (IQR)	10 (4–14)	12 (8–15)	0.02	0.066(−0.521 to 1.748)^a^	0.29	0.643(−0.579 to 1.865)^a^	0.30
GCS score at discharge degree, *n* (%)			0.05	0.638 (0.355–1.146)^b^	0.13	1.390 (0.836–2.312)^b^	0.21
3-8	55 (44.00)	25 (28.09)		NA	NA	NA	NA
9–12	26 (20.80)	21 (23.60)		NA	NA	NA	NA
13–15	44 (35.20)	43 (48.31)		NA	NA	NA	NA
mRS score at discharge, median (IQR)	4 (3–5)	4 (3–5)	0.15	1.183 (0.689–2.029)^b^	0.54	1.053 (0.641–1.729)^b^	0.84
mRS 0–1, *n* (%)	2 (1.60)	2 (2.25)	>0.99*	0.856 (0.039–18.703)^c^	0.92	1.590 (0.183–13.852)^c^	0.67
mRS 0–2, *n* (%)	7 (5.60)	6 (6.74)	0.73	0.896 (0.212–3.784)^c^	0.88	1.190 (0.371–3.814)^c^	0.77
Mortality in hospital, *n* (%)	12 (9.60)	4 (4.49)	0.16	0.489 (0.121–1.980)^c^	0.32	0.524 (0.162–1.692)^c^	0.28
mRS score at 3 months, median (IQR)	5 (3–6)	4 (3–6)	0.01	1.785 (1.034–3.081)	0.04	1.498 (0.913–2.457)^b^	0.11
mRS 0–1 at 3 months, *n* (%)	5 (4.00)	4 (4.49)	>0.99*	0.766 (0.157–3.734)^c^	0.74	0.980 (0.239–4.028)^c^	0.98
mRS 0–2 at 3 months, *n* (%)	21 (16.80)	16 (17.98)	0.82	0.939 (0.393–2.243)^c^	0.89	0.828 (0.394–1.740)^c^	0.62
Mortality within 3 months, *n* (%)	56 (45.90)	24 (26.97)	0.01	0.458 (0.225–0.931)^c^	0.03	0.519 (0.290–0.929)^c^	0.03
mRS score at 6 months, median (IQR)	5 (3–6)	4 (2–6)	0.01	1.924 (1.099–3.366)^b^	0.02	1.555 (0.945–2.556)^b^	0.08
mRS 0–1 at 6 months, *n* (%)	17 (13.60)	15 (16.85)	0.51	1.387 (0.570–3.372)^c^	0.47	1.165 (0.535–2.538)^c^	0.70
mRS 0–2 at 6 months, *n* (%)	27 (21.60)	24 (26.97)	0.36	1.139 (0.521–2.489)^c^	0.74	1.020 (0.531–1.960)^c^	0.95
Mortality within 6 months, *n* (%)	59 (48.36)	26 (29.21)	0.01	0.467 (0.233–0.936)^c^	0.03	0.544 (0.307–0.963)^c^	0.04
mRS score at 1 year, median (IQR)	5 (3–6)	3 (2–6)	0.01	2.031 (1.154–3.575)^b^	0.01	1.611 (0.977–2.654)^b^	0.06
mRS 0–1 at 1 year, *n* (%)	18 (14.40)	17 (19.10)	0.36	1.260 (0.521–3.050)^c^	0.61	1.080 (0.513–2.274)^c^	0.84
mRS 0–2 at 1 year, *n* (%)	29 (23.20)	27 (30.34)	0.24	1.412 (0.660–3.022)^c^	0.37	1.140 (0.607–2.140)^c^	0.68
Mortality within 1 year, *n* (%)	61 (50.00)	27 (30.34)	0.004	0.451 (0.226–0.901)^c^	0.02	0.530 (0.300–0.937)^c^	0.03

Adjusted variable: age, sex, hypertension, GCS, ICH volume, Coronary heart disease, surgery procedure during time(m), ICH location. GCS, Glasgow Coma Scale; mRS, Modified Rankin Scale; IQR, interquartile range; and NA, not applicable. Adjusted model and IPTW model taking the following variables into account: coronary heart disease, GCS score at admission, hematoma volume, site of hematoma, and surgery procedure during time(m). *Indicated the comparison between two groups using Fisher’s exact test. ^a^The β values were estimated from a linear regression. ^b^The common odds ratios were estimated from a ordinal logistic regression. ^c^The odds ratios were estimated from a binary logistic regression.

### Subgroup analyses

**Figure 4:**
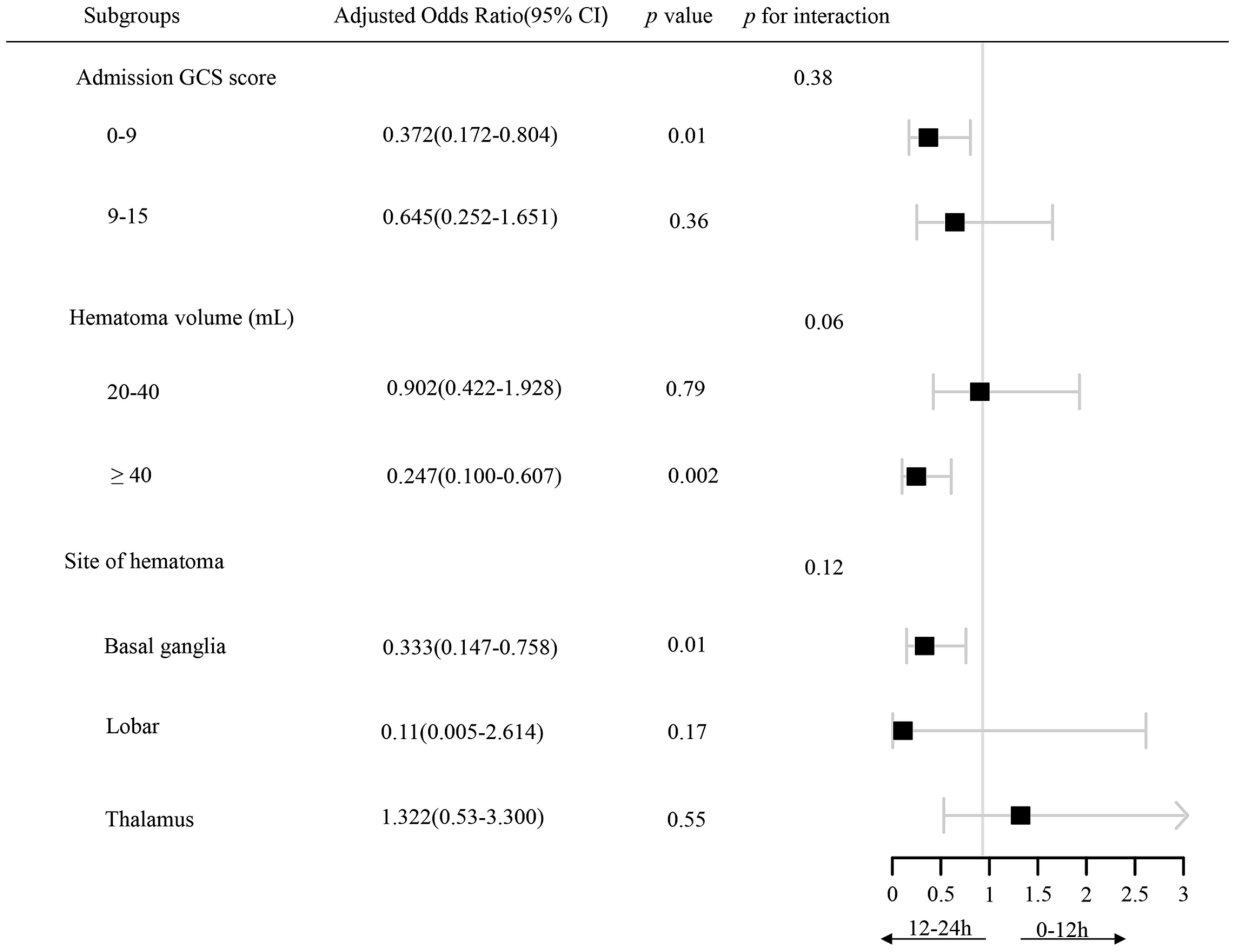
Forest plot illustrating the association between time to evacuation and long-term mortality across different subgroup cohorts. All statistical analyses were based on an IPTW model, accounting for the following variables: age, sex, hypertension, coronary heart disease, GCS score at admission, hematoma volume, site of hematoma, and surgical procedure during the time (m). GCS refers to the Glasgow Coma Scale.

Our study conducted subgroup analyses based on admission GCS score (0–9 score and 9–15 score), hematoma volume (20–40 mL and ≥40 mL), and hematoma location (basal ganglia, lobar, and thalamus) in Figure 4. Among patients with a low GCS score (0–9), the 12–24 h group was associated with a decreased risk of mortality within 1 year [OR, 0.372; 95% CI (0.172–0.804), *p* = 0.01] in the IPTW model. For patients with a hematoma volume ≥40 mL, performing MIPD between 12 to 24 h after symptom onset was associated with a lower mortality rate [OR, 0.247; 95% CI (0.100–0.607), *p* = 0.002]. In patients with sICH in the basal ganglia region, the association between time to evacuation and mortality was also significant [aOR, 0.333; 95% CI (0.147–0.758), *p* = 0.01].

A subsequent analysis was conducted to explore the effect of time from onset to MIPD across different hematoma volumes (as shown in Supplementary Table S1). Short- and long-term mortality in patients who underwent MIPD between 12 and 24 h after symptom onset was lower than in those treated within 12 h. The proportion of favorable outcomes at 6 months and 1 year was lower in the 0–12 h group (18.18% vs. 27.27% at 6 months; 18.18% vs. 29.09% at 1 year), although the differences were not statistically significant.

## Discussion

This study comprehensively assessed the association between time to evacuation and clinical outcomes in patients who underwent MIPD due to sICH, using multiple analytical methods. For patients treated within 24 h after acute sICH, undergoing MIPD between 12 and 24 h after symptom onset was associated with a lower risk of mortality at 1 year compared to those treated within 0 to 12 h.

A prospective, non-randomized comparative study demonstrated that patients who underwent MIPD within 24 h had a higher proportion of neurological independence and a lower proportion of mortality at 1 year (8). Additionally, the STICH II trial confirmed that early surgery (hematoma evacuation within 12 h) did not increase the proportion of mortality or disability at 6 months (10). The Kaplan–Meier plot from the STICH II trial indicated that early surgery could prolong survival time, although no significant difference was observed. Overall, MIPD was performed to evacuate hematomas, benefiting patients with sICH. However, it remains unclear when the optimal time for evacuation is. A subgroup analysis of MISTIE III revealed that patients who underwent minimally invasive hematoma evacuation within 36 h after symptom onset had trends toward more favorable outcomes compared to later treatment [aOR, 2.02; 95% CI (0.91–4.48)] (7). It seems that the optimal treatment window lies within the first 24 h after hemorrhage onset (13, 14). Our study observed that patients who underwent MIPD within 12 h after symptom onset had a higher proportion of mortality compared to those treated between 12 and 24 h. Indeed, a multicenter, retrospective study also found that patients who underwent MIPD within 6 h had a higher mortality rate at 3 months compared to those treated later (11). One plausible explanation is the instability of the hematoma with ongoing bleeding during the shorter time interval (15). Ovesen et al. (16) published that significant hematoma expansion was observed within 12 h (*p* < 0.001), with the most robust expansion occurring 7 to 8 h after symptom onset. Hematomas play an important role in inhibiting rebleeding, thereby reducing the risk of secondary inflammatory injury and neurotoxic edema (17). Furthermore, early surgery to remove the hematoma could increase the risk of surgical complications and prolong procedure time. Morgenstern et.al (18) reported that hematoma evacuation within 4 h after symptom onset was associated with rebleeding. In contrast, a meta-analysis suggested that early surgery (within 8 h of surgery) was associated with a decreased risk of unfavorable outcomes [OR, 0.59; 95% CI (0.42–0.84)] (19). Selection bias may explain the discrepancy. The meta-analysis included the study by Wang et al. (20), which showed significant differences in the number of patients across different time windows from ictus to treatment (0–8 h: *n* = 144; 8–24 h: *n* = 30; 24–72 h: *n* = 20). In addition, differences in baseline characteristics may partly explain the higher mortality observed in the 0–12 h group. In our study, patients with more severe neurological deficits were more likely to undergo MIPD earlier (median GCS score: 7 in the 0–12 h group vs. 8 in the 12–24 h group, *p* = 0.01). A lower GCS score has been identified as an independent predictor of mortality in patients with ICH (21, 22). Furthermore, hematoma location is also associated with mortality, particularly in cases of brainstem hemorrhage. Brainstem hemorrhage often necessitates emergency surgery because rapid hematoma expansion and mass effect may compress vital centers responsible for consciousness, respiration, and circulation. In our cohort, brainstem hemorrhage was more common among patients who underwent MIPD within 6 h, which may have contributed to the higher mortality in this group (23, 24).

Interestingly, although no significant differences were observed in the proportions of favorable and excellent outcomes among the 0–6 h, 6–12 h, and 12–24 h groups, a longer interval from symptom onset to hematoma evacuation was associated with a lower risk of mortality. A previous study investigating the optimal time window for patients with ICH reported similar findings, showing no significant differences in neurological function among the 0–6 h, 6–12 h, and 12–24 h groups (10.1 vs. 9.0 vs. 9.5, *p* = 0.19) at the 2-week follow-up, as assessed by the Scandinavian Stroke Scale (11). Several possible explanations may account for this discrepancy between mortality and functional outcomes. Our analysis of mRS distributions at discharge and at 3, 6, and 12 months suggested that patients who underwent MIPD within 12 h tended to have worse functional status, as reflected by higher mRS scores (Supplementary Table S2) and a lower proportion of mRS 0–3 (Table S3). One possible explanation is that patients with more severe disability were more likely to die during follow-up, thereby attenuating observable differences in functional outcomes among survivors. In addition, the limited sample size may have reduced the statistical power to detect significant differences in functional outcomes; therefore, these findings should be interpreted with caution.

The interaction between time to evacuation and hematoma volume may be more critical in acute therapeutic trials for sICH than in those for ischemic stroke (25). Interestingly, among patients with a hematoma volume greater than 40 mL, later surgery resulted in lower mortality at 1 year, according to the subgroup analysis. Therefore, patients were further stratified by hematoma volume to explore the effect of time to evacuation, as shown in Supplementary Table S1. For patients with hematoma volumes exceeding 40 mL, undergoing MIPD between 12 and 24 h after symptom onset was associated with better outcomes compared to those treated within 0–12 h. Hematoma volume is a significant predictor of mortality in patients with sICH (26, 27). Xiao et al. (11) reported that among patients with a hematoma volume of 30–50 mL, 3-month mortality was higher in those who underwent surgery within 0–6 h (23.8%) compared to those in the 6–12 h (11.3%) and 12–24 h (9.9%) groups (*p* = 0.042). In our findings, the 3-month mortality rates for the 0–6 h, 6–12 h, and 12–24 h groups were 46.7, 54.3, and 23.6%, respectively. The mortality in our study was relatively high, which may be explained by the severe neurological deficits observed at admission (the median GCS score at admission in our study was 8, compared to 10.8 in Xiao et al.). Additionally, it seems reasonable to suggest that medium hematoma volume plays a role in limiting hematoma expansion, which is an independent predictor of worsened mortality and morbidity (28).

There are several limitations to this study. First, the retrospective nature of the study could introduce unavoidable selection bias; however, we used statistical methods, including multinomial logistic regression and IPTW, to mitigate its potential impact. Second, some data were not collected, including residual hematoma volume, anesthesia time, blood loss, pulmonary infections, and epilepsy. Third, the study was retrospective, conducted at two centers, and had a relatively small sample size in the 0–6 h group, which may have contributed to the lack of significant differences in functional recovery. However, despite the absence of statistical significance, there was a trend suggesting that patients who underwent MIPD within 12 to 24 h after symptom onset had a positive effect on favorable outcomes at 1 year. Due to the observational nature of this study, residual time-to-evacuation bias could not be completely excluded. Although we adjusted for several clinically relevant confounders, including disease severity and surgical procedure, unmeasured or inadequately captured factors may still have influenced both treatment timing and mortality. Therefore, the association between time from onset to MIPD and mortality should be interpreted with caution.

## Conclusion

Among patients with sICH, a slight prolongation of time to MIPD was associated with a decreased risk of mortality at 1 year. Our findings suggest that undergoing MIPD within 12 to 24 h after symptom onset may be beneficial. Future clinical trials or pooled analyses with larger sample sizes of MIPD patients are needed to further confirm these results.

## Data Availability

The data analyzed in this study is subject to the following licenses/restrictions: Access to datasets may be obtained by sending an email request to the corresponding author. Requests to access these datasets should be directed to 18192071686@163.com.
