# Supplementary material for: Long-term mortality and time to minimally invasive puncture and drainage in spontaneous intracerebral hemorrhage
Source: Front Neurol. 2026 Apr 29;17:1829539. doi: 10.3389/fneur.2026.1829539 (PMC13167574; doi:10.3389/fneur.2026.1829539)
Supplement: Supplementary file 1 [file Table_1.DOCX]

sTable1. Comparison of clinical outcomes following early versus delayed MIPD, stratified by hematoma volume

|  | 20-40ml (n=108) | | | ≥40 ml (n=106) | | |
| --- | --- | --- | --- | --- | --- | --- |
|  | <12 | 12-24 | P value | <12 | 12-24 | P value |
| Rebleeding, n (%) | 5(6.76) | 2(5.88) | >0.99* | 2(3.92) | 0 | 0.30 |
| GCS score at discharge, median (IQR) | 10(5-14) | 11(4-15) | 0.45 | 10(3-14) | 13(9-15) | 0.03 |
| GCS score at discharge degree, n (%) |  |  | 0.68 |  |  | 0.09 |
| 3-8 | 35(47.30) | 14(41.18) |  | 20(39.22) | 11(20.00) |  |
| 9-12 | 15(20.27) | 6(17.65) |  | 11(21.57) | 15(27.27) |  |
| 13-15 | 24(32.43) | 14(41.18) |  | 20(39.22) | 29(52.73) |  |
| mRS score at discharge, median (IQR) | 4(3-5) | 4(3-5) | 0.85 | 4(4-5) | 4(3-5) | 0.10 |
| mRS 0-1, n (%) | 2(2.70) | 1(2.94) | >0.99* | 0 | 1(1.82) | >0.99* |
| mRS 0-2, n (%) | 5(6.76) | 2(5.88) | >0.99* | 2(3.92) | 4(7.27) | 0.68* |
| Mortality in hospital, n (%) | 5(6.76) | 2(5.88) | >0.99* | 7(13.73) | 2(3.64) | 0.09 |
| mRS score at 3 months, median (IQR) | 5(3-6) | 4(3-6) | 0.51 | 6(4-6) | 4(3-5) | 0.01 |
| mRS 0-1 at 3 months, n (%) | 3(4.05) | 1(2.94) | >0.99* | 2(3.92) | 3(5.45) | >0.99* |
| mRS 0-2 at 3 months, n (%) | 13(17.57) | 8(23.53) | 0.47 | 8(15.69) | 8(14.55) | 0.87 |
| Mortality within 3 months, n (%) | 30(41.67) | 11(32.35) | 0.36 | 26(52.00) | 13(23.64) | 0.003 |
| mRS score at 6 months, median (IQR) | 5(3-6) | 4(1-6) | 0.44 | 6(3-6) | 3(2-5) | 0.003 |
| mRS 0-1 at 6 months, n (%) | 10(13.51) | 9(26.47) | 0.10 | 7(13.73) | 6(10.91) | 0.66 |
| mRS 0-2 at 6 months, n (%) | 18(24.32) | 9(26.47) | 0.81 | 9(17.65) | 15(27.27) | 0.24 |
| Mortality within 6 months, n (%) | 32(44.44) | 13(38.24) | 0.55 | 27(54.00) | 13(23.64) | 0.001 |
| mRS score at 1 year, median (IQR) | 5(2-6) | 4(1-6) | 0.31 | 6(3-6) | 3(2-6) | 0.003 |
| mRS 0-1 at 1 year, n (%) | 10(13.51) | 9(26.47) | 0.10 | 8(15.69) | 8(14.55) | 0.87 |
| mRS 0-2 at 1 year, n (%) | 20(27.03) | 11(32.35) | 0.57 | 9(17.65) | 16(29.09) | 0.17 |
| Mortality within 1 year, n (%) | 33(45.83) | 13(38.24) | 0.46 | 28(56.00) | 14(25.45) | 0.001 |

MIPD, minimally invasive puncture and drainage; GCS, Glasgow Coma Scale; mRS, Modified Rankin Scale; and IQR, interquartile range.

* indicated the comparison among three groups using Fisher's exact test.

sTable2. The distribution of mRS score in patients underwent MIPD stratified by time to evacuation

| mRS | mRS at discharge | | mRS at 1 months | | mRS at 3 months | | mRS at 6 months | | mRS at 1 year | |
| --- | --- | --- | --- | --- | --- | --- | --- | --- | --- | --- |
|  | <12h | 12-24h | <12h | 12-24h | <12h | 12-24h | <12h | 12-24h | <12h | 12-24h |
| 1 | 1.60% | 2.25% | 2.46% | 2.25% | 4.10% | 4.49% | 13.93% | 16.85% | 14.75% | 19.10% |
| 2 | 4.00% | 4.49% | 2.46% | 3.37% | 13.11% | 13.48% | 8.20% | 10.11% | 9.02% | 11.24% |
| 3 | 19.20% | 20.22% | 13.11% | 21.35% | 8.20% | 16.85% | 11.48% | 22.47% | 9.84% | 21.35% |
| 4 | 28.00% | 37.08% | 19.67% | 24.72% | 18.03% | 30.34% | 10.66% | 16.85% | 9.84% | 14.61% |
| 5 | 37.60% | 31.46% | 17.21% | 22.47% | 10.66% | 7.87% | 7.38% | 4.49% | 6.56% | 3.37% |
| 6 | 9.60% | 4.49% | 45.08% | 25.84% | 45.90% | 26.97% | 48.36% | 29.21% | 50.00% | 30.34% |

sTable3. Proportion of patients with mRS 0–3 at 1-year follow-up

|  | <12h | 12-24h | p value |
| --- | --- | --- | --- |
| mRS 0-3 at discharge, n (%) | 31(24.80) | 24(26.97) | 0.72 |
| mRS 0-3 at 1 month, n (%) | 22(17.60) | 24(26.97) | 0.10 |
| mRS 0-3 at 3 months, n (%) | 31(24.80) | 31(34.83) | 0.11 |
| mRS 0-3 at 6 months, n (%) | 41(32.80) | 44(49.44) | 0.01 |
| mRS 0-3 at 1 year, n (%) | 41(32.80) | 46(51.69) | 0.01 |
